# Emotion Regulation Difficulties Are Not Always Associated With Negative Outcomes on Women: The Buffer Effect of HRV

**DOI:** 10.3389/fpsyg.2020.00697

**Published:** 2020-04-30

**Authors:** Carole Fantini-Hauwel, Elise Batselé, Cassandra Gois, Xavier Noel

**Affiliations:** ^1^Department of Psychology, Research Center for Clinical Psychology, Psychopathology and Psychosomatics, Université libre de Bruxelles, Brussels, Belgium; ^2^Department of Medicine, Psychological Medicine Laboratory, Faculty of Medicine, Université libre de Bruxelles, Brussels, Belgium

**Keywords:** emotion regulation, heart rate variability, HRV, depression, vagal control

## Abstract

The Heart Rate Variability (HRV) is regularly associated with depression and trait emotion regulation. However, the interaction between HRV and emotional disturbances is still debated. Only a few studies indicate that HRV moderates the effect of personality traits involved in psychopathological disorders. Since the regulation of emotions is a transdiagnostic factor for most psychological disorders, this study aimed to explore whether HRV moderates the relationship between trait emotion dysregulation and depressive symptoms. We collected data from 148 participants via online questionnaires and HRV measurements at rest. Results show for the 114 female remaining in the study that whereas high emotion regulation difficulties led to higher depressive symptoms severity when resting HRV is low, depressive symptoms remain stable in the same condition but when resting HRV is high. Overall, high resting HRV appears to dampen the consequences of trait emotion regulation difficulties. Further studies are needed to confirm this result, but this suggests that usual response tendencies could be overcome by deactivating or inhibitory processes such as those implied in cognitive flexibility reflected through HRV, according to the neurovisceral integration model.

## Introduction

The relationship between depression and heart rate variability (HRV) stems from observations that cardiac patients with depression were more prone to adverse outcomes. Indeed, depression is considered to be an important and independent risk factor predicting cardiac mortality, the occurrence of more intense cardiac events, and having been implicated in mid-term survival (Carney et al., 2005; Frasure-Smith and Lespérance, 2008). The underlying question was about the potential common mechanism between depression and heart health to explain why and how mood disorders had this negative effect. It has been suggested that HRV is one of the key potential factors (Carney et al., 2002, 2005).

HRV reflects the evolution of cardiac activity to meet the demands of the environment by indexing the characteristics of the autonomic nervous system (ANS, Malik et al., 1996) and the homeostasis between sympathetic and parasympathetic activity. Importantly, under resting conditions, the length variations between heartbeat intervals (HRV) are under the cardiac vagal control which is prevalent (Malik et al., 1996). In this context, HRV is generally termed “vagally mediated-HRV” (we will refer to resting vagally mediated-HRV throughout the text), which is particularly important regarding psychopathology (Beauchaine and Thayer, 2015). HRV is widely considered as an index of the ability to regulate emotions (Appelhans and Luecken, 2006). Indeed, emotional experience is associated with physiological arousal based on the ANS which is subdivided into a sympathetic nervous system pathway (SNS, Excitatory) and a parasympathetic nervous system pathway (PNS, inhibitor). SNS activity increases during emotional events leading to an accelerated heart rate while the PNS inhibits its own activity (vagal brake) to allow rapid adaptation to these demanding events. During a non-challenging situation, the PNS is responsible for lower arousal (low excitation) and then of a decelerated heart frequency. The interplay between SNS and PNS allows an important modulation of emotional responses and their physiological manifestations, depending on the context, referring to autonomous flexibility (Appelhans and Luecken, 2006). This flexibility is indexed through a varying length of heartbeats intervals representing the HRV. At rest, an imbalance between these two branches in favor of the SNS and leads to a decrease of the ANS flexibility and, consequently, to a reduction of the HRV.

Some studies have shown that HRV at rest was associated with a higher risk of death after myocardial infarction (for a review, see Buccelletti et al., 2009) but especially for our purposes, depression was an independent risk factor of adverse medical outcomes in coronary patients (Lichtman et al., 2014), through biobehavioral mechanisms including HRV (Carney and Freedland, 2017). Indeed, a meta-analysis supports an association between reduced HRV, particularly a decrease in PNS activity, and depression; i.e., the HRV decreases with increasing severity of depression (Kemp et al., 2010). This negative relationship was also found in a meta-analysis focused on young people across clinical versus healthy populations (Paniccia et al., 2017). However, there are also some inconsistencies in the literature (Moser et al., 1998; Hammel et al., 2011). Even if the relation between HRV at rest and depression was significant, it has been demonstrated that this relation was mainly and more importantly driven by some antidepressant drugs (tricyclic generally, Licht et al., 2008; Kemp et al., 2014), depending of the time domain-HRV measure considered (Licht et al., 2008). Indeed, Licht et al. (2008) evidenced that the association between depression and HRV was fully mediated by medication (except for selective serotonin reuptake inhibitor) when considering SDNN (Standard Deviation of Normal to Normal interval), a measure reflecting both SNS and PNS activity. This association remains significant, albeit to a lesser extent when considering RMSSD (Root Mean Square of Standard Deviation) reflecting the PNS activity. However, this is also controversial as it has been noticed that antidepressants did not influence HRV in a study investigating depressive symptoms evolution, leading the authors to consider resting-HRV as a vulnerability biomarker of mood disorders (Brunoni et al., 2013). Nonetheless, this does not apply to unmedicated people. This biomarker vulnerability has also been suggested in a longitudinal study where low resting-HRV participants, without clinically relevant depressive symptoms at first stage, were more likely to develop clinically relevant depressive symptoms or take antidepressants 10 years later, even after controlling for potential confounders (Jandackova et al., 2016). These results suggest that HRV precedes the onset of depressive symptoms severity and not the other way around. Finally, the lack of consensus regarding the relation between HRV and depression could also be related to specific characteristics of depression such as the melancholic feature (Borrione et al., 2018), suggesting that depression measures could influence this association with regard to the weight of some items. Anyway, a recent meta-analysis has confirmed that unmedicated depressed patients were lower on HRV than controls (Koch et al., 2019). Further, in a non-clinical sample, depression symptoms were higher when HRV was lower (Paniccia et al., 2017).

All in all, studies about the relation between resting-HRV and depression or depressive symptoms suggest that potential variables could moderate this association. Among those, emotion regulation is an important process to relate depression and HRV as both of them have been associated with difficulties to regulate negative emotions.

Past studies have largely emphasized the crucial role of emotion regulation in many disorders (for review, see Aldao et al., 2010) leading it to be considered a key factor underlying psychopathology. Thompson (1994) defined emotion regulation as “the extrinsic and intrinsic processes responsible for monitoring, evaluating, and modifying emotional reactions, especially their intensive and temporal features, to accomplish one’s goals”. The construct of emotion regulation could be theorized as a set of strategies, i.e., reappraisal, suppression… (Gross, 1998) or, more broadly, as an overall deficit in emotional functioning and regulation implying (a) the awareness, understanding, and acceptance of emotional experiences; (b) the ability to engage in goal-directed behaviors and inhibit impulsive behaviors when experiencing negative emotions; (c) the flexible use of situationally appropriate strategies to modulate the intensity and/or duration of emotional responses; and (d) the willingness to experience negative emotions as part of pursuing meaningful activities in life (Gratz and Roemer, 2004). Many studies have highlighted that depression level or depression vulnerability was associated with an impairment in the use of emotion regulation strategies such as reappraisal, expressive suppression, rumination (Ehring et al., 2010; Joormann and Gotlib, 2010) or an overall impairment affecting both the experience (acceptance, awareness, understanding) and the modulation of the emotional experience (Brockmeyer et al., 2012). Further, a recent meta-analysis evidenced that interventions targeting strategies or overall functioning were followed by a decrease of the symptomatology of affective disorders, including depression (Sloan et al., 2017).

Resting-HRV is thought to be a transdiagnostic biomarker of psychopathology (Beauchaine and Thayer, 2015) and potentially a vulnerability marker of depression. However, it is actually considered as a biomarker of top-down self-regulation including both behavioral and emotional regulation, without specific self-regulatory processes (Holzman and Bridgett, 2017). Thus, it appears that the HRV represents top-down ER abilities in a broad sense and not a narrow one, reflecting executive functioning, inhibitory control, attentional regulation and working memory (Hansen et al., 2003; Thayer et al., 2009). As psychopathology reflects a dysregulation of positive and negative emotions, investigating the relations between trait-like ER and HRV is important. There is an increasing number of studies investigating the relation between ER and resting-HRV. There is a large consensus about the negative relation between resting-HRV and ER, the lower resting-HRV is, the higher are ER difficulties (Geisler et al., 2010; Visted et al., 2017 (for review, see Balzarotti et al., 2017; Holzman and Bridgett, 2017). Some studies didn’t evidenced such an association and sometimes showed the opposite results. However, these studies relied on ER strategies, i.e., suppression and reappraisal (Koval et al., 2013), measure resting-HRV during a paced breathing and not a spontaneous one (Butler et al., 2006) or during the view of a meditation film (Fabes and Eisenberg, 1997). It also has been shown that higher resting-HRV was related to less coherence between facial expression and self-reported experience; facial expression being attenuated while the experience was similar to those of low resting-HRV participants (Pu et al., 2010). These authors, nevertheless, maintained that high resting-HRV is an indicator of good ER abilities. They argued that high resting-HRV people are more inclined to spontaneously and automatically suppress facial expression when this is socially appropriate, and display a clear ability to regulate emotion when appropriate (flexible ER), which is not the same as people usually using emotional suppression.

Most studies hypothesized the relationship between HRV and psychological distress is mediated by ER (Geisler et al., 2010), following the neurovisceral integration model (Thayer and Lane, 2000) or the polyvagal theory (Porges, 2001), as HRV and emotion regulation both involve prefrontal cortical areas. However, it seems difficult to exclude a potential moderation effect. Although executive functioning is closely related to HRV because of shared underlying neural circuits, one study shows that executive functioning moderates the relationship between depression and HRV so that depression predicts a lower level of HRV among participants presenting with low executive functioning (Gathright et al., 2016). More interestingly, it has also been shown that the consequences of thought suppression efforts, an emotion regulation strategy, are more likely to occur at a low HRV level, but not at a high level. Indeed, an increased spontaneous suppression effort predicted greater persistence of unwanted thoughts, then more distress only when HRV is weak. As the HRV increases, the strength of this relation decreases (Gillie et al., 2015), suggesting that the HRV could also be related to the cognitive control capacities and mitigate the negative effects of suppression of thought. However, studies examining the moderator effect of HRV are very rare and generally do not take into account the overall process for regulating self-perceived emotion. The objective of this study was to evaluate the extent to which self-perceived ER difficulties and resting-HRV contribute to depressive symptoms. As described above, the HRV could be considered to reflect, at least partially, the effectiveness of the executive functions involved in the regulation of emotions. Self-perception of ER reflects what people think about how they usually manage emotional encounters that do not necessarily reflect what people really do in the present moment (state response). State responses depend on contextual issues that will or will not allow cognitive and behavioral resources to sustain the achievement of objectives. We hypothesize that the relationship between ER difficulties and depressive symptoms is moderated by HRV with an effect exacerbated by ER difficulties at a low HRV level.

## Materials and Methods

### Participants

This study is part of a larger research program focused on emotion regulation, emotional consciousness, heart rate variability, and psychopathology.

A total of 148 participants took part to the experiment. They were recruited via an advertisement on the university campus and no incentives were given. The study was conducted by a clinical psychologist (EB) who encountered all participants and asked all socio-demographical data, medical background, as well as current or past psychopathological disorders and drug consumption. Any participant indicating past or actual chronic or psychiatric disorders, or taking any treatment was discarded to obtain the more healthier sample as possible.

Exclusion criteria were: (1) having a chronic somatic disease, (2) having a psychiatric diagnosis, or (3) having difficulties understanding the French language. 6 participants were discarded due to somatic or psychiatric exclusion criteria exclusion (anxiety, irritable bowel, cystic fibrosis, anorexia, hypotension, diabetes). Thus, 142 participants remained in the study. However, 11 subjects were then excluded from the analysis due to missing data or physiological recording problems. Thus, the final sample was composed of 131 students (114 females, mean age: 20.83 ± 1.99, age range: 18–28) from which we discarded one outlier. As there were only 17 males in the sample, we discarded them for all analysis as gender should interact with HRV. The resulting sample was 114 females. The study was approved by the faculty ethics committee, and no incentive was given.

### Questionnaires

#### Self-Perceived Emotion Regulation Difficulties

ER difficulties were assessed using the French version of the Difficulties in Emotion Regulation Scale-Short Form (DERS-SF; Kaufman et al., 2016). The DERS-SF comprises 18-items and six sub-scales designed to measure different facets of ER difficulties. Participants were asked to respond on a scale from 1 (*almost never*) to 5 (*almost always*). The subscales include: (1) non-acceptance of emotional responses (α = 0.80, “When I’m upset, I become embarrassed for feeling that way”), (2) difficulties engaging in goal-oriented behaviors when experience negative emotions (α = 0.86, “When I’m upset, I have difficulty getting work done”), (3) difficulties in controlling impulsive behaviors when experiencing negative emotions (α = 0.89, “When I’m upset, I have difficulty controlling my behaviors”), (4) lack of emotional awareness (α = 0.80, “When I’m upset, I acknowledge my emotions”), (5) lack of strategies to regulate emotions (α = 0.77, “When I’m upset, I believe that wallowing in it is all I can do”), and (6) lack of emotional clarity (α = 0.56, “I am confused about how I feel”). The total score exhibited good internal consistency with α = 0.84 and means that when scores increase, difficulties in ER are more important.”

#### Depressive Symptomatology

We used here the depression subscale of the French version of the Hospital Anxiety and Depression Scale (Zigmond and Snaith, 1983). This 7-items subscale is scored on a 4-point Likert scale ranging from 0 to 3. The internal consistency was satisfying with α = 0.72). The more scores are high, the more depressive symptoms are important.

#### Resting-HRV Measure

The resting HRV was measured by using a Polar^®^ V800 heart rate monitor allowing to extract HRV parameters. The measure taken by the Polar^®^ V800 has been validated as comparable to an electrocardiograph (Giles et al., 2016). The electrode belt was dampened and placed, following Polar’s guidelines, tightly but comfortably just below the chest muscles. Resting measurements were conducted in a sitting position for 5 min, according to the recommendations of the Task Force of the European Society of Cardiology and The North American Society of Pacing and Electrophysiology (1996). The variability between successive R-spikes (or variability within inter-beat-intervals. IBIs) was directly extracted with Polar Flow in a text file and then analyzed using Kubios HRV analysis package 3.0.2 premium version (Tarvainen et al., 2014) allowing for the calculation of time-domain indices of HRV. Artifacts within the R-R series were visually detected to select a period of 5 consecutive minutes relatively artifact free. We applied then an automatic artifact correction level. In the automatic correction algorithm, artifacts are detected from dRR series (differences between successive RR intervals), a robust and validated way to detect ectopic and misplaced beats from normal beats (Lipponen and Tarvainen, 2019). To separate ectopic and normal beats, time-varying threshold is used where ectopic beats are corrected by interpolated RR values. Missed beats and extra beats are corrected by adding new R wave or removing extra R wave occurrence time, RR interval series being recalculated.

We used Root Mean Square Standard Deviation (RMSSD) time frequency parameter as it indexes parasympathetic activity without being influenced by sympathetic one and recognized to be more free of respiration rate during resting influence (Hill et al., 2009). The HRV index we used in this study has been natural log-transformed (ln) to fit the assumptions of linear analyses (Ellis et al., 2008). By convention, we will use the term HRV throughout the text to refer to RMSSD.

### Procedure

All participants were instructed not to smoke, take physical exercise, or drink coffee/alcohol/energizing drinks three hours before going to the experience. They were placed in a soundproof experimental room and were given a detailed explanation of the procedures that would take place without indicating the specific hypothesis under the study. Demographical data were also gathered during this familiarization period and responses to participants’ questions were provided. Electrode belt was attached to the subject’s chest to complete a 5-min resting HRV where participants, while spontaneously breathing (no instruction was given about breathing), were sat in front of a blank screen and were instructed not to move or fall asleep. The measure started 3-4 minutes after there were sat for a duration of 7 min. After the HRV measure, participants were asked to complete a set of online questionnaires. The total duration of the study was approximately 30 minutes.

### Statistical Analyses

We have examined Pearson’s correlations between all variables. We ran hierarchical linear regressions with HRV and ER difficulties in step 1, and the interaction between HRV and ER difficulties in step 2. All Independent variables were mean-centered and the interaction term was computed by crossing the mean centered IV of interest following Aiken and West (1991). In case of significant interaction, high and low values for the moderator variable were derived using ±1 SD from the mean and simple slopes analysis were conducted. All statistical tests were conducted using STATA 14.

## Results

Descriptive statistics are provided in Table 1.

**TABLE 1:** Descriptive statistics.

Variable	Mean	Standard Deviation	Min	Max
Age	20.79	2.01	18	28
Awareness	6.93	2.52	3	15
Clarity	6.64	2.36	3	12
Goals	9.66	3.36	3	15
Impulse	7.15	3.21	3	15
Non-acceptance	6.96	2.84	3	15
Strategies	7.95	3.01	3	15
DERS total scores	45.23	10.94	21	75
Depression	3.84	2.78	0	13
Ln-HRV	3.59	0.49	2.33	4.72

### Pearson’s Correlations

HRV was significantly and negatively associated with depressive symptoms and DERS total scores as well as with all DERS dimensions except “Awareness,” “clarity,” and “non-acceptance.” The higher is heart rate variability, the lower are emotion regulation difficulties and depression. As attended, the DERS total scores and all dimensions except “non-acceptance” and “awareness” were positively related to depression. More specifically, individuals who experience more difficulties to meet goals when facing negative emotions, more difficulties in controlling impulses, a lack of strategies to regulate emotions, and a lack of emotion clarity, were those who have more depressive symptoms. All bivariate correlations are given in Table 2.

**TABLE 2:** Pearson’s correlations.

Variables	Depression	HRV
Depression	–	−0.20*
Awareness	0.15	0.08
Clarity	0.32***	–0.17
Goals	0.21*	−0.23**
Impulse	0.43***	−0.22*
Non-acceptance	–0.02	–0.09
Strategies	0.37***	−0.25**
DERS total score	0.39***	−0.28**
HRV	−0.20*	–

***p < *0.001*, **p < *0.01*, *p < *0.05.*

### Hierarchical Moderation Analyses

All detailed coefficients are given in Table 3.

**TABLE 3:** Standardized coefficients of the Hierarchical regressions predicting depression by HRV, Emotion regulation difficulties and HRV X Emotion regulation difficulties interaction.

	DERS		Non-acceptance		Awareness		Clarity		Goals		Impulse		Strategies	
	β (SE)	η^2^	β (SE)	η^2^	β (SE)	η^2^	β (SE)	η^2^	β (SE)	η^2^	β (SE)	η^2^	β (SE)	η^2^
RMSSD	**−0.07(0.51)**	0.01												
DERS	0.34***(0.02)	0.11												
RMSSD X DERS	−0.20*(0.05)	0.05												
RMSSD			−0.20*(0.54)	0.04										
Non-acceptance			0.04(0.09)	0.00										
RMSSD X non-acceptance			−0.04(0.19)	0.00										
RMSSD					−0.19*(0.53)	0.04								
Awarenesss					0.13(0.10)	0.02								
RMSSD X awareness					−0.03 (0.26)	0.00								
RMSSD							**−0.14(0.51)**	0.02						
Clarity							0.27**(0.11)	0.07						
RMSSD X clarity							**−0.17^*t*^(0.22)**	0.03						
RMSSD									**−0.13(0.55)**	0.02				
Goals									0.20*(0.08)	0.04				
RMSSD X Goals									**−0.13(0.17)**	0.02				
RMSSD											**−0.09(0.51)**	0.01		
Impulse											0.37***(0.08)	0.13		
RMSSD X Impulse											**−0.13(0.17)**	0.02		
RMSSD													**−0.11(0.51)**	0.01
Strategies													0.32***(0.08)	0.11
RMSSD X Strategies													−0.19*(0.17)	0.04
R	**0.20**		0.04		0.06		**0.15**		**0.09**		**0.21**		**0.19**	
R^2^	**0.18**		0.02		0.03		**0.13**		**0.06**		**0.19**		**0.16**	
F(3, 110)	9.01***		1.65		2.20		6.41***		3.40*		9.67***		8.31***	

***p ≤ 0.001, **p ≤ 0.01, *p ≤ 0.05, ^*t*^trend to significance.

#### Emotion Regulation Difficulties Total Score (Figure 1)

The overall model explained 20% of the depressive symptoms variance [*F*(3, 109) = 9.01, *p* ≤ 0.001]. The DERS total scores significantly contribute to the depression scores (η^2^ = 0.11) as well as the HRV (η^2^ = 0.01) but in a lesser extent. The contribution of the interaction effect was 4% [*F*(1, 109) = 5.29, *p* ≤ 0.05]. The simple slopes analysis show that when HRV is −1 SD below the mean, the effect of ER difficulties was significant [b = 0.14, t = 4.51, *p* ≤ 0.001, 95% BCI (0.08 to 0.20)] while at +1 SD above the mean, the effect was not [b = 0.03, t = 0.84, ns, 95% BCI (−0.04 to 0.10)].

**FIGURE 1:**
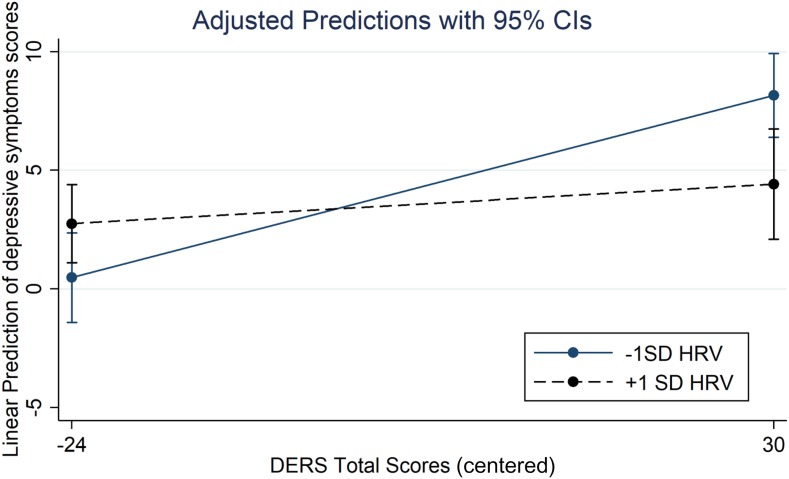
Interaction between HRV and Emotion regulation difficulties on depression scores.

To better understand which ER abilities were moderated by HRV, we have run the same model for all subdimensions of the ER difficulties scale.

#### Awareness

The overall model was non-significant [*F*(3, 109) = 2.20, *p* = 0.09, *R*^2^ = 0.06].

#### Goals

The overall model was significant [*F*(3, 109) = 3.40, *p* ≤ 0.05, *R*^2^ = 0.09]. We only found a main effect of Goals (η^2^ = 0.04).

#### Impulse

We found a significant effect of impulse (η^2^ = 0.13) on depressive symptoms, the overall model explaining 21% of the variance [*F*(3, 109) = 9.67, *p* ≤ 0.001]. HRV and the interaction were non-significant.

#### Clarity

The overall model explained 15% of the depressive symptom scores variance [*F*(3, 109) = 6.41, *p* ≤ 0.001]. There was a main effect of clarity (η^2^ = 0.07) and a trend to significance for the interaction between clarity and HRV.

#### Strategies

We found a main effect of strategies (η^2^ = 0.11) and a significant interaction between Strategies and HRV (η^2^ = 0.05) explaining 4% [*F*(1, 109) = 4.64, *p* ≤ 0.05] of the overall model [19%, *F*(3, 109) = 8.31, *p* ≤ 0.001]. The simple slopes analysis evidenced that at −1 SD below the HRV mean, the effect of strategies was significant [*b* = 0.49, t = 4.29, *p* ≤ 0.001, 95% CI (0.26 to 0.71)] but not at +1 SD [b = 0.11, t = 0.89, ns, 95%CI (−0.14 to 0.36)].

#### Non-Acceptance

The overall model was not significant [*F*(3, 109) = 1.65, *p* = 0.18].

## Discussion

The purpose of the study was to examine the extent to which ER difficulties and the vagal control contribute to depressive symptoms. Specifically, to what extent the relationship between ER difficulties and the severity of depressive symptoms was potentially influenced by vagal control.

Our results are consistent with those obtained earlier because a lower HRV was associated with more ER difficulties (Gerardo et al., 2016; Visted et al., 2017).

More importantly, it appears that HRV moderates the relationship between ER difficulties and depressive symptoms. At a higher level of HRV, ER difficulties are not related to depressive symptom scores, whereas at a low HRV level, ER difficulties were associated with increased depression symptom scores. Thus, a high HRV seems to mitigate the effect of self-perceived ER difficulties, especially when individuals perceive they lack strategies to regulate their own emotions. This result is in line with some studies demonstrating the buffering effect of HRV on negative emotions (Fabes and Eisenberg, 1997; Dywan et al., 2008). It has also been shown that the deleterious effect of expressive suppression (an ER strategy) on negative emotional experiences was observed only at a low level of resting-HRV during a conflict between partners (Geisler and Schröder-Abé, 2015). In the same vein, the negative consequences of suppression of thinking occur primarily in people with low HRV, but not in those who are elevated (Gillie et al., 2015). These studies suggest a buffer effect of HRV on individual differences involved in negative outcomes. This is also consistent with a study showing that the effect of neuroticism was inhibited at a higher level of HRV on the outcomes of daily life (Ode et al., 2010). Finally, Yaroslavsky et al. (2013) showed that individuals with high resting-HRV felt less distressed after a maladaptive response, such as ruminative thinking, than individuals with low resting-HRV. However, these studies relied on ER strategies and our findings extend these results to ER functioning.

Several directions could be proposed to explain our results that investigate ER process and functioning rather than ER strategies. First, the DERS scale evaluates dispositional tendencies and not state-based dysregulation (Lavender et al., 2017) or, in other words, self-perceived emotion regulation difficulties measure behavioral disposition and not necessarily actual abilities. Habitual ERs have been shown to correlate only moderately with what individuals actually do in a stressful task (Egloff et al., 2006), suggesting that contextual and personally relevant triggers should overcome dispositional tendencies and that other processes could help to disable or inhibit habitual tendencies, such as those involved in cognitive flexibility. According to the neurovisceral integration model, HRV is an indicator of the prefrontal cortex functioning involving many cognitive control processes to achieve personal goals (Thayer and Lane, 2000; Thayer et al., 2009). Of these, the HRV reflects the strength of inhibitory and attentional control, two key elements for successful ER (Park et al., 2013; Park and Thayer, 2014; Ottaviani et al., 2018). Inhibitory control allows the suppression of prepotent responses while attentional control allows engaging or disengaging from relevant stimuli. The former has been shown to disrupt the relationship between ER difficulties (difficulties to access to effective ER) and distress tolerance. ER difficulties did not predict distress tolerance in individuals who have more attentional control while those with lower levels exhibited less distress tolerance (Bardeen et al., 2015). Thus, these processes underpin what we call cognitive flexibility, which has been associated with HRV in a task-switching paradigm (Colzato et al., 2018), where a higher resting-HRV predicts better switching performances.

Second, self-reported measures are more likely to reflect perceived rather than actual level of ER difficulties because they require good self-awareness or insight onto our own abilities to accurately report them (Ciarrochi et al., 2001). We cannot, therefore, exclude the fact that people with high self-perceived ER difficulties have, in fact, accurate ER abilities when they have greater HRV, if we consider that the HRV reflects the emotion regulation based on the performance.

This study suggests a potential buffering effect of the vagally mediated-HRV on negative emotions when individuals feel they have difficulties with their emotion regulation functioning. However, some limitations must be reported. Our population is a convenient population and the range of depressive symptom scores is limited. Further, a semi-structural clinical interview could have provided more accurate information about potential psychopathological disorders. So, this result should be replicated with individuals who have been diagnosed with a major depressive disorder to ensure that HRV also buffers any emotional regulation deficit. This study is correlational and does not support a strong conclusion on the buffering effect and indicates that focusing on this aspect should be relevant. Gender differences have also been frequently reported in the literature, so replicating this study using a sample of women and men is warranted. Finally, visual examination of inter-beats intervals for artifacts was not possible as data were not recorded with ECG. However, regarding the HRV quantification, the kubios’ robust algorithm accuracy detection of missed, extra-beats and misaligned-beats was 99.3% for simulated abnormal beats and 96.96% for real abnormal beats (Lipponen and Tarvainen, 2019).

The future perspective involves replicating this study in an experimental emotional induction plan to determine if an HRV buffering effect in a challenging situation is really present, and to take into account a potential joint effect of resting-HRV and phasic HRV (reactivity). In conclusion, our result may suggest that HRV augmentation through clinical interventions such as biofeedback (Caldwell and Steffen, 2018), mindfulness (Burg et al., 2012) or executive functioning enhancement (Xiu et al., 2016) can be an additional perspective to help reduce depressive symptoms when people cannot disengage them from what they usually do to regulate their emotional states.

## Data Availability Statement

The datasets analyzed in this article are not publicly available. Requests to access the datasets should be directed to CF-H, carole.fantini@ulb.be.

## Ethics Statement

The studies involving human participants were reviewed and approved by the Faculty of Psychology Ethic Committee, ULB. The patients/participants provided their written informed consent to participate in this study.

## Author Contributions

CF-H: study design experiment, writing, and statistical analysis. XN: statistical analysis and supervision of the final version of the draft. CG and EB: study design experiment, participant recruitment, and reviewing of the draft.

## Conflict of Interest

The authors declare that the research was conducted in the absence of any commercial or financial relationships that could be construed as a potential conflict of interest.
